# Clinical and Functional Characterization of a Missense *ELF2* Variant in a CANVAS Family

**DOI:** 10.3389/fgene.2018.00085

**Published:** 2018-03-23

**Authors:** Hena Ahmad, Teresa Requena, Lidia Frejo, Marien Cobo, Alvaro Gallego-Martinez, Francisco Martin, Jose A. Lopez-Escamez, Adolfo M. Bronstein

**Affiliations:** ^1^Division of Brain Sciences, Imperial College, Charing Cross Hospital, London, United Kingdom; ^2^National Hospital for Neurology & Neurosurgery, London, United Kingdom; ^3^Otology and Neurotology Group CTS495, Department of Genomic Medicine, Centro de Genómica e Investigación Oncológica (GENYO), Pfizer-Universidad de Granada-Junta de Andalucía, Granada, Spain; ^4^Gene and Cell Therapy Group, Department of Genomic Medicine, Centro de Genómica e Investigación Oncológica (GENYO), Pfizer-Universidad de Granada-Junta de Andalucía, Granada, Spain; ^5^Department of Otolaryngology, Instituto de Investigación Biosanitaria ibs.GRANADA, Hospital Virgen de las Nieves, Universidad de Granada, Granada, Spain

**Keywords:** cerebellar ataxia, vestibular hypofunction, neuropathy, whole-exome sequencing, ETS domain

## Abstract

Cerebellar ataxia with neuropathy and bilateral vestibular areflexia syndrome (CANVAS) is a rare disorder with an unknown etiology. We present a British family with presumed autosomal dominant CANVAS with incomplete penetrance and variable expressivity. Exome sequencing identified a rare missense variant in the *ELF2* gene at chr4:g.140058846 C > T, c.10G > A, p.A4T which segregated in all affected patients. By using transduced BE (2)-M17 cells, we found that the mutated ELF2 (mt-ELF2) gene increased ATXN2 and reduced ELOVL5 gene expression, the causal genes of type 2 and type 38 spinocerebellar ataxias. Both, western blot and confocal microscopy confirmed an increase of ataxin-2 in BE(2)-M17 cells transduced with lentivirus expressing mt-ELF2 (CEE-mt-*ELF2*), which was not observed in cells transduced with lentivirus expressing wt-ELF2 (CEE-wt-ELF2). Moreover, we observed a significant decrease in the number and size of lipid droplets in the CEE-mt-*ELF2*-transduced BE (2)-M17 cells, but not in the CEE-wt-ELF2-transduced BE (2)-M17. Furthermore, changes in the expression of ELOVL5 could be related with the reduction of lipid droplets in BE (2)-M17 cells. This work supports that ELF2 gene regulates the expression of ATXN2 and ELOVL5 genes, and defines new molecular links in the pathophysiology of cerebellar ataxias.

## Introduction

The triad of cerebellar ataxia, bilateral vestibulopathy, and peripheral neuropathy occurs between 9 and 32% of patients with bilateral vestibular failure (Bronstein et al., 1991; Zingler et al., 2007). It is a rare disorder termed Cerebellar ataxia with neuropathy and bilateral vestibular areflexia syndrome (CANVAS; [MIM: 614575]). A review reported 51 patients seen over a 10-year period (Szmulewicz et al., 2015), in agreement with our own estimates of seeing 6–8 new cases per year.

Cerebellar ataxia with neuropathy and bilateral vestibular areflexia syndrome is a late-onset, slowly progressive multi-system ataxia likely secondary to a neurodegenerative ganglionopathy. The combination of cerebellar ataxia and vestibular impairment produces a characteristic oculomotor sign of impaired (“broken up”) visually enhanced vestibulo-ocular reflex (VVOR) (Migliaccio et al., 2004). Phenotypic heterogeneity in CANVAS patients is recognized (Szmulewicz et al., 2014b). Although most cases are sporadic, the finding of six affected siblings’ pairs (Szmulewicz et al., 2014a) suggests a familial recessive disorder or a dominant inheritance with incomplete penetrance; however, the genes involved have not been elucidated.

### Case Presentation

**FIGURE 1:**
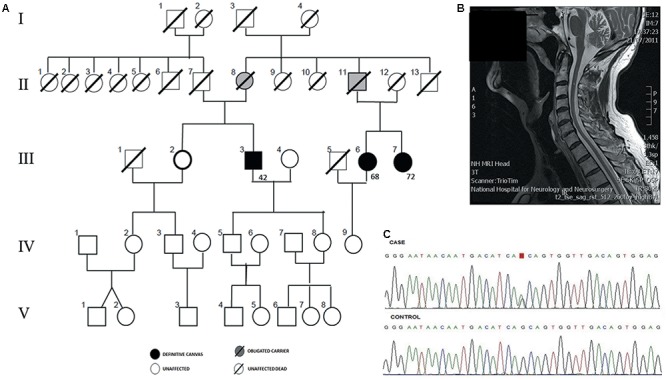
Genetic diagnosis of familial Cerebellar ataxia with neuropathy and bilateral vestibular areflexia syndrome (CANVAS). **(A)** Pedigree of an autosomal dominant CANVAS family with three affected cases with the age of onset. **(B)** Sagittal MRI showing cerebellar atrophy in patient III:3. **(C)** Chromatogram of reverse chain of the variant chr4:g.140058846 G > A from an affected individual (III.3) is compared to the sequence from a familial control (III.2).

We describe a non-consanguineous family with three CANVAS patients from England (**Figure 1A**). Genetic testing excluded Friedreich ataxia and SCA 1, 2, 3, 6, 7, and 38 as potential diagnoses. All patients provided written informed consent for their participation for publication and the study protocol was approved by the institutional review board. Family members in the fourth generation were examined and remained asymptomatic; however, symptom onset is typically delayed and usually over 60 years of age.

**FIGURE 2:**
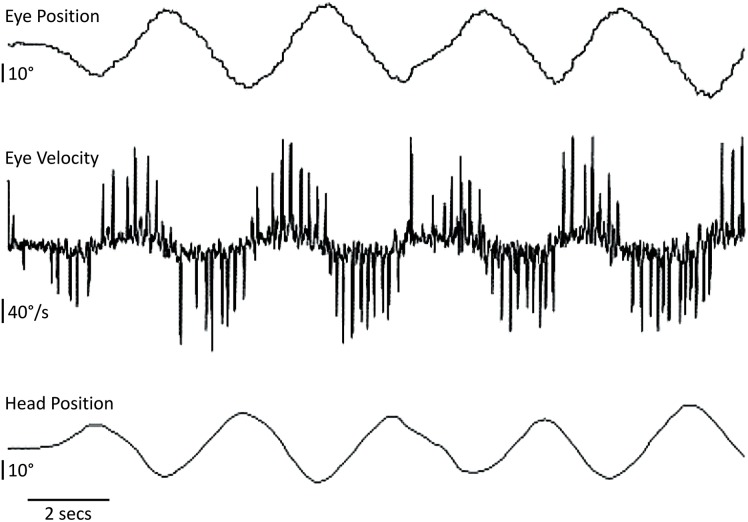
Head and eye horizontal movements in the CANVAS proband. The patient fixates a visual target on the wall while the examiner manually oscillates his head from behind in a quasi-sinusoidal fashion [visually assisted vestibulo-ocular reflex or visually enhanced vestibulo-ocular reflex (VVOR)]. The compensatory eye movement elicited is severely broken-up or cog-wheeled due to the presence of multiple eye saccades (best seen as ‘spikes’ in the eye velocity trace). Upward deflections correspond to rightward head or eye movements.

Patient III:3 (proband), was a 78 year old gentleman with 20 years of progressive loss of sensation distally in upper and lower limbs and a gradual deterioration in his balance. He developed oscillopsia in 2005 and in 2014 he noticed mild slurred speech and incoordination followed by development of a prominent dry cough, difficulty with micturition and erectile dysfunction. Examination revealed dysarthria, ataxic gait, and a positive Romberg test. Eye movement examination revealed downbeat nystagmus on lateral gaze. Smooth pursuit was broken horizontally and vertically. Saccades were moderately hypometric. The doll’s head-eye maneuver was abnormally jerky, with numerous “catch-up” saccades [abnormal VVOR; (**Figure 2**)]. Horizontal and vertical head impulse tests (HITs) were positive bilaterally. The rest of the cranial nerve examination was normal. Limb examination revealed normal tone and power throughout with no spasticity or extrapyramidal features. Reflexes were symmetrically present in the upper limbs, however, in the lower limbs, ankle jerks were absent and plantar were mute. There was a distal loss to light touch and pinprick sensation in all limbs, vibration sense was absent to the sternum with proprioceptive loss to ankles bilaterally. There was moderate bilateral upper and lower limb dysmetria. Romberg’s test was positive. Normal blood tests included negative anti-neuronal, anti-GAD, coeliac antibodies, anti-treponemal, paraneoplastic antibodies normal B1, B12, glucose, thyroid function, Mg, and vitamin E. Bithermal caloric and rotational electronystagmography confirmed bilateral absence of vestibular function. Nerve conduction study (NCS) revealed an axonal sensory neuronopathy. Sural nerve and muscle biopsy were normal. Autonomic function tests were normal. MRI brain showed cerebellar atrophy particularly involving the vermis (**Figure 1B**). The patient was diagnosed with CANVAS. His father (II:7) died of presumed stroke in his 60’s and his mother remained well until she died at the age of 96. Although the clinical record did not report any known neurological condition, II:8 was considered to be an obligated carrier. On further exploring the family history, it was discovered that III:6 and III:7 (maternal cousins of proband) had similar symptoms hence were also assessed. Of note, their father (II:11) had a balance disorder of unknown etiology therefore may have been affected.

Patient III:6, was a 78 year old lady with a 10 years history of slowly progressive imbalance, distal numbness, and dysesthesia. Over the last year she described dysphagia and occasional cough. Eye movement examination revealed an abnormal VVOR with HIT showing catch up saccades to the left. Pursuit movements were moderately broken up but in keeping with age. There was distal loss to pinprick in upper and lower limbs. Ankle reflexes were absent. She had an ataxic gait and Romberg’s was mildly positive. Bithermal caloric testing and rotational test (velocity steps and sinusoidal oscillation), showed significant bilateral reduction of vestibular function. Video-HIT showed consistent abnormal catch up saccades bilaterally. EMG/NCS confirmed a sensory neuronopathy. Autonomic function tests were normal. MRI brain revealed an incidental frontal cavernoma and mild global atrophy. This was in keeping with a diagnosis of incomplete (*‘forme fruste’*) CANVAS phenotype.

Patient III:7, was a 74 years old lady with a 2 years history of imbalance, especially in the dark, followed by distal neuropathic symptoms and severe coughing ‘fits.’ She denied any facial numbness or paresthesiae, speech or swallowing disturbance. Examination revealed a weak downbeat nystagmus in lateral gaze. Pursuit was broken in all directions and saccades were mildly hypometric. She had an abnormal VVOR and bilateral positive HIT. Reflexes were diminished throughout and ankle jerks were absent. There was distal sensory loss to light touch and pinprick in upper and lower limbs, proprioceptive impairment to wrists and ankles. Finger-nose testing was mildly impaired in upper limbs. She had a broad based ataxic gait and Romberg’s was positive. Investigations including cerebellar screening, blood tests, and genetic tests were normal. Autonomic function tests were normal. Bilateral vestibular hypofunction was confirmed on caloric and rotational test. EMG/NCS confirmed axonal sensory neuronopathy with absent sensory nerve action potentials. MRI brain showed fissural prominence within the superior cerebellar vermis. A cervical spine MRI showed a slender lower cervical/upper thoracic cord with flattening of the posterior surface and faint signal change dorsally, compatible with dorsal root ganglionopathy. These features represent a typical CANVAS phenotype.

The fourth subject (III: 2) was a 73 years old lady without any neurological symptoms and a normal neurological examination.

### Whole-Exome Sequencing

We sequenced the exomes of four individuals in the family (III:3, III:6, III:7, and III:2) (**Figure 1A**). Exons capture, library preparation and sequencing were performed as we previously described, in a SOLiD 5500xl platform using the reference sequence GRChr37/hg19 (Martin-Sierra et al., 2016). Only variants were considered. Single nucleotide variants (SNVs) with coverage >30X and minor allele frequency (MAF) <0.001 were retrieved using a combined filtering strategy (Requena et al., 2017). Variants found in the non-affected sibling (III:2) (**Figure 1C**), were discarded and 3622 variants were retained for further analyses. ANNOVAR software was used to annotate and filter SNVs. Finally, 30 heterozygous SNVs remained after filtering by exome data from the Exome Aggregation Consortium, 1000 Genomes databases and in-house controls. Twenty-seven SNVs had been previously annotated and three of them were novel variants. We also used LOD scores derived from WES-common SNVs to reduce the list of candidate variants, as previously described (Gazal et al., 2016), and 10 candidate variants remained (Supplementary Tables S1, S2). The selected candidate variant, a missense heterozygous variant in the coding regions of *ELF2* [NM_201999.2], that segregated with the phenotype was validated by Sanger sequencing. The candidate variant has been submitted to ClinVar database^1^.

We searched for rare variants in the *ELF2* gene in exome sequencing datasets from two additional British CANVAS families, and we also performed Sanger sequencing of the *ELF2* gene in these two families and a third one from Spain. So, a total of eight additional unrelated individuals with CANVAS were sequenced, however, none of them carried the variant or other rare variants in the coding regions of *ELF2.*

The rare variant leads to a change in the exon 2 of the transcript sequence (p.A4T). The predicted effect on protein function is probably damaging, since the beginning of the coding sequence is highly conserved across species and matches with the protein N-terminal elf transcription factor domain, encoded from 4th residue to 108th residue (Supplementary Figures S1, S2). At protein level, the elf-2 amino acids sequence has a 67 and 57% of positive homology matches with elf-1 and ets-1, respectively. The known ETS-binding domain has 87% homology among the three transcription factors (TFs), and the amino acid (p.A4) is conserved in the sequence of *ETS-1*, *ELF-1,* and *ELF-2* (Supplementary Figure S3). A PAVIVE motif on N-terminal elf transcription factor domain, a relevant recognition motif in elf family, is conserved between elf-1 and elf-2 amino acids sequences.

### BE(2)-M17 Cell Culture

Human neuroblastoma BE(2)-M17 cell line (ATCC^®^ CRL-2267^TM^) was cultured and RT-PCR was used to confirm that the *ELF2, ATXN2,* and *ELOV5L* genes are constitutively expressed in BE(2)-M17 neuroblastoma cell line (Supplementary Figures S4A,C).

### Lentiviral Vector Constructs Production and Neuroblastoma Transduction

The cDNA encoding for human *ELF2* gene and the *ELF2* gene with the variant described was cloned in the bicistronic lentiviral vector (LV) pHRSINcppt_CMVeGFP_ELF1α-TetR (also named CEET, available in our laboratory) using standard molecular biology techniques [PacI/MreI (Sse232I)] to obtain the lentiviral plasmids CEE-wt-*ELF2* and CEE-mt-*ELF2*, respectively. Both LV expressed eGFP in addition to the *wt-ELF2* or the *mt-ELF2*. LVs production was performed as previously described (Frecha et al., 2008). All the LVs used were titrated based on the percentage of eGFP expressing cells as previously described (Benabdellah et al., 2014).

The transduction efficiency was 95%. The number of LV integrated per cell was estimated by qRT-PCR as previously described (Cobo et al., 2013). Transduction was measured at 3, 7, 10, and 25 days). No significant differences were found between both transduced cell lines. Moreover, the transduction remained stable over time after day 3 (Supplementary Figure S4B).

### Cell Viability and Proliferation Assays

Cell viability and proliferation assays were performed in BE(2)-M17 cells to investigate the effect of the *ELF2* variant. For both cell viability and proliferation assays, there was no difference between the cells. (Supplementary Figure S5). These results suggest that overexpression of wt-*ELF2* or *mt-ELF2* gene did not have any influence on the proliferation or survival of BE(2)-M17 cells and overexpression of *ATXN2* did not modify the morphology.

### Functional Assays: qRT-PCR, Western Blot, Immunocytochemistry, and Confocal Microscopy

We also investigated the effect of mutant *ELF2* on *ATNX2* and *ELOVL5* expression levels, since these genes are a direct target of *ELF2,* according to Curated Transcription Factor Targets Dataset (TRANSFAC), and both have been associated with SCA2 and SCA38 (Scoles et al., 2012; Di Gregorio et al., 2014; Hoxha et al., 2017).

**FIGURE 3:**
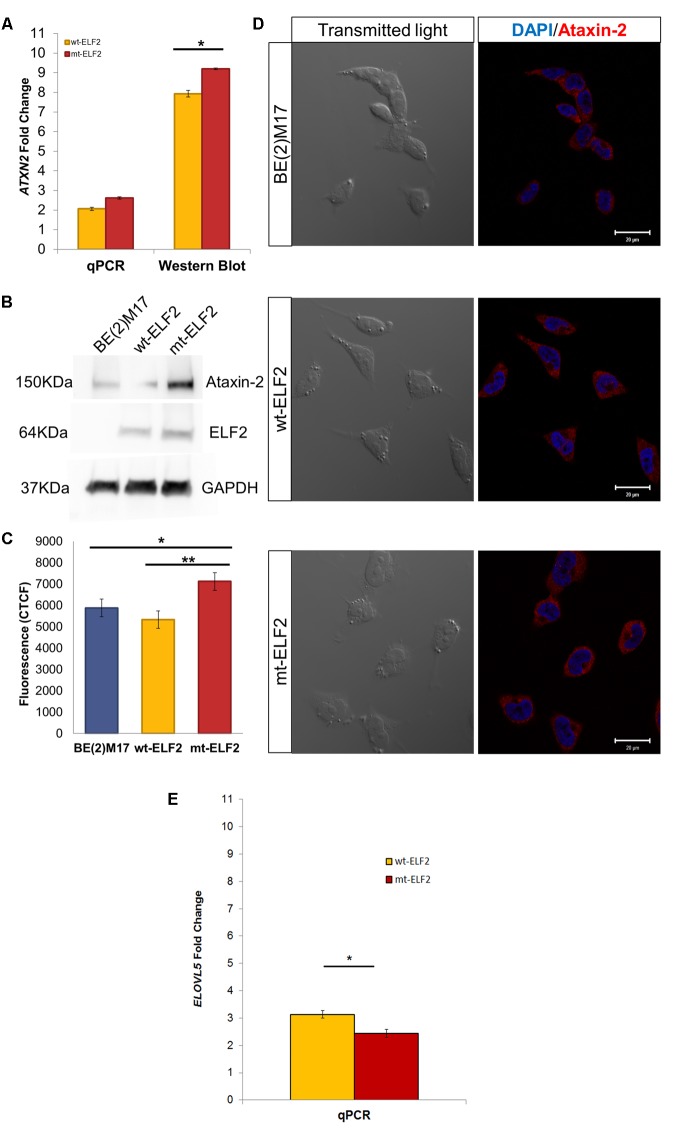
*ATXN2* expression in BE(2)M17, wt-ELF2 and mt-ELF2 transduced cells. **(A)** ATXN2 qPCR and ataxin-2 Western blot show statistical differences between wt-*ELF2* and mt-*ELF2* transduced cells, both in qPCR and Western blot. **(B)** Representative western blot of BE(2)M17 exhibiting an increased content of ATXN2 in mt-*ELF2* transduced cells. ATXN2 (#611378, 1:1000), Elf2 (#HPA006057-100UL, 1:1000), GAPDH (#AB2302, 1:3000 and secondary antibodies #HAF007, 1:6000, #HAF008, 1:3000, #A9046-1ML, 1:10000. **(C)** CTCF emitted by BE(2)M17 cells labeled with anti-ataxin-2 antibody in non-transduced, wt-*ELF2* transduced and mt-ELF2 cells. **(D)** Representative immunocytochemistry image of ataxin-2 in non-transduced BE(2)M17, wt-*ELF2,* and mt-*ELF2* transduced cells showing an increased staining in mt-*ELF2* cell-line. ^∗^*p* < 0.02, ^∗∗^*p* < 0.002. Primary antibodies anti-ataxin-2 (1:250) and anti-ELF2 (1:500) and visualized with Alexa-555-conjugated goat anti-mouse #A-21422, 1:500 and Alexa-633-conjugated goat anti-rabbit #A-21071, 1:500, respectively. **(E)** *ELOVL5* qPCR show statistical differences between wt-*ELF2* and mt-*ELF2* transduced cells. ^∗^*p* < 0.003.

We confirmed that *ELF2*, *ATXN2*, and *ELOVL5* genes were constitutively expressed in BE(2)-M17 cells by RT-PCR. We then evaluated *ELF2,* *ATXN2*, and *ELOVL5* gene expression in CEE-wt-*ELF2*- and CEE-mt-*ELF2*-transduced BE(2)-M17 cells by qPCR and Western blot and found a significant increase in both *ELF2* (*p* = 0.03) and *ATNX2* (*p* = 0.002) expression at mRNA levels in the cells transduced with the CEE-mt-*ELF2*, but not in cells transduced with the CEE-wt-*ELF2* (**Figure 3A**). In contrast, *ELOVL5* was significantly decreased (*p* = 0.003) in cells transduced with the CEE-mt-*ELF2*, but not in cells transduced with the CEE-wt-*ELF2* (**Figure 3E**). The *ATXN2* increase was confirmed at protein levels in the CEE-mt-*ELF2*-transduced BE(2)-M17 cells, when they were compared to the wild type cell line (*p* = 0.019, **Figures 3A,B**).

Confocal microscopy imaging illustrated an overexpressed cytoplasmic distribution of ataxin-2 in CEE-mt-*ELF2*-transduced BE(2)M17 cells. We quantified the fluorescence intensity levels (**Figure 3D**). CEE-mt-*ELF2* cell line was the most intensely labeled, followed by those cells that were not transduced and finally wt-*ELF2* cells. Significant differences were found among non-transduced cells compared to mt-*ELF2* (*p* = 0.03) and between wt-*ELF2* as compared to mt-*ELF2* (*p* = 0.003, **Figure 3C**). In addition, the immunocytochemistry showed that the transduction and mutation did not change elf2 location.

**FIGURE 4:**
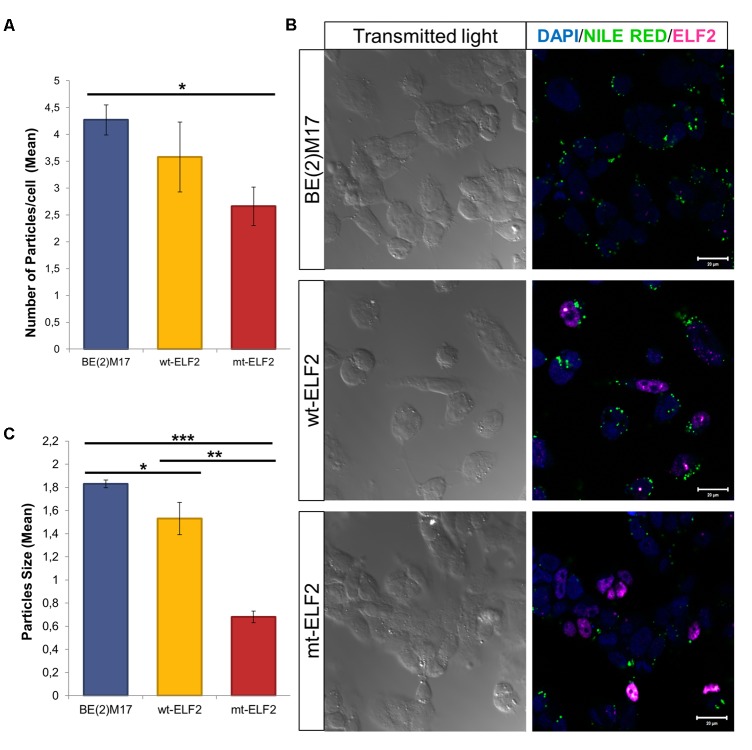
Changes in Lipid droplets in transduced BE(2)M17 cell-lines. **(A)** Number of lipid droplets particles per cell in each cell-line (^∗^*p* = 0.02). **(B)** Representative immunocytochemistry image of Lipid droplets stained with Nile Red in non-transduced BE(2)M17, *wt-ELF2* and *mt-ELF2* transduced cells showing a decrease number and size of the droplets in *mt-ELF2* cell-line. For lipid droplets experiments, cells were stained with Nile red to measure the number and size of lipid droplets. After Nile red staining, cells were fixed and staining with anti-ELF2 (1:500) and visualized with Alexa-633-conjugated goat anti-rabbit (1:500). **(C)** Mean of particles size in every cell-line. BE(2)M17 non-transduced cells vs. *wt-ELF2* cells (^∗^*p* = 0.03); *wt-ELF2* vs. *mt-ELF2* transduced cells (^∗∗^*p* = 1.54 × 10^-8^); BE(2)M17 vs. *mt-ELF2* (^∗∗∗^*p* = 1.55× 10^-48^).

On comparing non-transduced BE(2)M17 cells with CEE-mt-*ELF2* BE(2)M17-transduced cells, significant differences in the number of lipid droplets were observed with reduced lipid droplets present in the mutant cell line (*p* = 0.02, **Figures 4A,B**). In addition, we observed that lipid droplets were smaller in CEE-*mt-ELF2* transduced BE(2)M17 cells (0.68 ± 0.05) when compared with CEE-*wt-ELF2* BE(2)M17 transduced cells (1.53 ± 0.14, *p* = 1.54 × 10^-8^) and non-transduced BE(2)M17 cells (1.83 ± 0.03, *p* = 1.55 × 10^-48^, **Figure 4C**).

## Background

The *ETS* gene family is a group of TFs divided in 12 subfamilies. The *ETS* subfamily includes *ETS1* and *ETS2*; the ELF subfamily includes *ELF1, ELF2*, and *ELF4* (MEF) genes and the ELG subfamily consist of *GABPα*. All ETS TFs are defined by a highly conserved DNA binding domain, the ETS domain with a core GGA(A/T) DNA sequence (Sharrocks, 2001). Previous electrophoretic mobility shift assays (EMSAs) have demonstrated that ETS1, ELF2, and GABPα interact with the ETS domain within the 5′-UTR in the *ATXN2* gene in HEK293 and SH-SY5Y nuclear lysates. HEK293 cells overexpressing ETS1 showed an increase in the expression of *ATXN2* gene (Scoles et al., 2012). These findings suggested that the ETS domain in *ATXN2* may be regulated by other TFs of the ETS gene family such as *ELF-2*. In the present study, we identified a novel missense variant in the *ELF2* gene (E74-like factor 2; *NERF*), which segregates the complete phenotype and we present functional data showing the effect of mutated *ELF2* (mt-ELF2) gene on *ATXN2* and *ELOVL5* two genes previously associated with spinocerebellar ataxia 2 and 38 (SCA2 and SCA38). No similar phenotype has been linked to ELF2 mutations at the time of submission (see section “Concluding Remarks”).

## Discussion

Cerebellar ataxia with neuropathy and bilateral vestibular areflexia syndrome is a rare syndrome, with less than 500 cases described worldwide (Szmulewicz et al., 2015), and familial cases have been described rarely (Szmulewicz et al., 2014a). We report a family with three CANVAS patients segregating a novel variant in *ELF2* gene. Several lines of evidence support a pathogenic role for the *ELF2* variant in this family. Firstly, multiple bioinformatics tools ranked this variant at the top of the candidate list; secondly, this novel variant was not found in the gnomAD and, perhaps more conclusively, the mt-ELF2 in a neuroblastoma cell line was able to modify the gene expression of two genes associated with ataxia in two ways. Firstly, by upregulating the expression and translation of *ATXN2* (the gene involved in SCA2) and secondly, by decreasing the expression and translation of *ELOVL5*, (associated with SCA 38). Sequencing data were re-evaluated in our familial dataset in both genes, but no abnormal CAG repeat expansion in *ATXN2* or pathogenic variants in *ELOVL5* gene such as c.214C>G or c.689G>Tl were found in the patients.

ELF2 is a TF associated with RUNX1 and both interact in the regulation of gene expression (Wang et al., 1993). We have observed that ELF2 acts as a repressor of *ATXN2* gene expression in neuroblastoma cells and that mt-*ELF2* will not be likely to regulate its expression. Although our mutation is not within the ETS-binding domain, it is not possible to exclude the interaction of ELF2 and other TFs, such as RUNX1.

*ELOVL5* is a target gene for ELF2 according to the TRANSFAC (Wingender et al., 2000; Wingender, 2008) and this gene is considered the causal gene of SCA 38 (Di Gregorio et al., 2014). Our results also confirm that *mt-ELF2* also modifies the expression of *ELOVL5.* This gene is involved in the long-chain fatty acids elongation cycle, and it is highly expressed in Purkinje cells. Furthermore, the ELOVL5^-/-^ mice develop ataxia and motor impairment during the balance beam test (Hoxha et al., 2017). Several neurological diseases, particularly hereditary spastic paraplegias (Dick et al., 2010; Tesson et al., 2012; Boukhris et al., 2013; Martin et al., 2013) display alterations of lipid metabolism. Increases in lipid droplets play a crucial role in the nervous system and have been associated with *in vitro* models of neurodegenerative disorders such as Huntington’s and Parkinson’s diseases (Martinez-Vicente et al., 2010; Thiam et al., 2013; Welte, 2015), emphasizing the importance of lipid homeostasis in brain membranes.

Although the expression of *ELF2* gene in the human cerebellum is low according to the Allen Brain Atlas^2^ (Hawrylycz et al., 2012), and the same variant was not observed in other CANVAS patients, this may be attributed to the genetic heterogeneity commonly found in hereditary ataxias.

Furthermore, we have found strong evidence that the position chr4:g.140058846 C > T in the *ELF2* gene is highly conserved in an evolutionary sense, therefore the variant is likely pathogenic and possibly interferes with protein function. Functional assays indicate a regulatory role of the *ELF2* variant *in vitro* for two SCA genes, since we have shown that the expression of mt-*ELF2*, but not wt-*ELF2*, increases *ATXN2* gene expression and ataxin-2 translation and decreases *ELOVL5* gene expression in BE(2)-M17 cells.

## Concluding Remarks

We describe a rare variant in *ELF2* gene in this family with CANVAS syndrome and demonstrate its functional effects in *ATXN2* and *ELOV5* genes in BE(2)-M17 transduced cells. The interaction between *ELF2,* *ATXN2*, and *ELOVL5* genes found suggests that the regulation of expression in these genes could potentially be a shared mechanism in hereditary ataxias.

## Ethics Statement

This study was carried out in accordance with the recommendations of Imperial College Research Ethics Committee with written informed consent from all subjects. All subjects gave written informed consent in accordance with the Declaration of Helsinki. The protocol was approved by the Imperial College Research Ethics Committee.

## Author Contributions

HA, TR, LF, MC, AG-M, FM, JL-E, and AB substantially contributed to the conception and design of the work. AB and HA examined the patients. TR, LF, and MC carried out the lab experiments. AG-M and TR performed the bioinformatic analyses of NGS data. All authors analyzed and interpreted the data for the work, drafted the work, revised it critically for important intellectual content, finally approved the version to be published, and all agreed to be accountable for all aspects of the work in ensuring that questions related to the accuracy or integrity of any part of the work are appropriately investigated and resolved.

## Conflict of Interest Statement

The authors declare that the research was conducted in the absence of any commercial or financial relationships that could be construed as a potential conflict of interest. The reviewer GH and handling Editor declared their shared affiliation.

## Footnotes

1 http://www.ncbi.nlm.nih.gov/clinvar/

2 http://www.brain-map.org/
