# Supplementary material for: Clinical and Functional Characterization of a Missense ELF2 Variant in a CANVAS Family
Source: Front Genet. 2018 Mar 23;9:85. doi: 10.3389/fgene.2018.00085 (PMC5876245; doi:10.3389/fgene.2018.00085)
Supplement: Supplementary file 1 [file Table_1.pdf]

frontiers

Supplementary Material

Clinical and functional characterization of a missense ELF2 variant in a CANVAS family

Hena Ahmad.MD[1]. Teresa Requena. PhD[2]. Lidia Frejo[2]. Marien Cobo. PhD[3]. Alvaro Gallego-Martinez[2]. Francisco Martin. PhD[3]. Jose A Lopez-Escamez. PhD. MD[2.4.] *. Adolfo M. Bronstein. PhD.MD[1.] *

Correspondence:

a.bronstein@imperial.ac.uk

antonio.lopezescamez@genyo.es

Supplementary methods

qPCR. Quantitative RT-PCR (qPCR) was performed with Sybergreen kit and ABI 7900 HT Fast Real-Time PCR Systems (Life Technologies) following commercial instructions (40 cycles standard conditions) and the following primers Fw--> GTGGAGGTGTCAACTGAAGAGTC and Rv-->TACCTAACTCAGGAGACCCATTG that cover partially exon 5 and 6 that are expressed in all the isoforms. Hypoxanthine phosphoribosyltransferase 1 (HPRT-1) was used as a house keeping gene. Technical triplicate was performed per each sample was run in technical triplicate to reduce experimental errors. The fold change for each gene results were obtain analysed using the comparative CT method

Western-blot. Protein concentration was determined by Bradford protein assay (Bio-Rad. 15 µg of total proteins were separated for molecular weight in a poliacrilamyde gel (130V, 1h 30min) and transferred to a Trans-Blot® Turbo™ Midi PVDF membrane by Trans-Blot® Turbo™ Transfer System (BioRad). The membrane was incubated with primary antibodies overnight at 4ºC. A mouse monoclonal antibody against ATXN2 (BD Biosciences, #611378, 1:1000), a rabbit polyclonal antibody against Elf2 (Sigma-Aldrich, #HPA006057-100UL, 1:1000) and a chicken polyclonal antibody against GAPDH (EMD Millipore, #AB2302, 1:3000) were used. Then the membrane was incubated with secondary antibodies for 1h at room temperature. A goat anti-mouse (R&D Systems, #HAF007, 1:6000), a goat anti-rabbit (R&D Systems, #HAF008, 1:3000) and a rabbit anti-chicken (Sigma-Aldrich, #A9046-1ML, 1:10000) were used respectively. We developed the membrane using Clarity™ Western ECL Substrate (Bio-rad) and images were obtained by the Image Quant LAS4000 (GE Healthcare Life Science). We used ImageJ software (NIH Image) for protein quantification.

Immunocytochemistry. For lipid droplets experiments, cells were stained with 4 µg/mL Nile red in PBS (made from 1 mg/mL Nile red stock solution in acetone, Sigma-Aldrich) for 25 minutes under

dark conditions to measure the number and size of lipid droplets. After Nile red staining, cells were fixed with 4% paraformaldehyde fixative solution for 20 min, and then washed with 0.1M Glycine (Sigma-Aldrich) in 1x PBS (Sigma-Aldrich). Then, samples were incubated with 0.3% Triton TM X-100 (Sigma-Aldrich) in 1x PBS for 30 min at RT and washed with 1x PBS twice. Blocking was performed during 30 min in 1x PBS with 3% bovine serum albumin (BSA, Sigma-Aldrich). Anti-ELF2 (1:500) was used as a primary antibody and cells were incubated overnight in blocking solution (BSA 3%) and visualized with Alexa-633-conjugated goat anti-rabbit (1:500).

Cells were mounted in SlowFade® mounting medium with DAPI (Life technologies) for nuclei staining. A laser scanning confocal microscope LSM 710 (Carl Zeiss) was used for image collection and the Zeiss browser software program ZEN (black edition) was used to acquire and export the data. All images were taken with defined laser intensity settings on the microscope. Final image processing and labeling were performed with the image analysis program ImageJ.

Supplementary references

1. Oettgen P, Akbarali Y, Boltax J, Best J, Kunsch C, Libermann TA. Characterization of NERF, a novel transcription factor related to the Ets factor ELF-1. Mol Cell Biol 1996 Sep;16(9):5091–106.

2. Pan, X., Wilson, M., McConville, C., Brundler, M.A., Arvanitis, T.N., Shockcor, J.P., Griffin, J.L., Kauppinen, R.A. and Peet, A.C. (2012) The lipid composition of isolated cytoplasmic lipid droplets from a human cancer cell line, BE(2)M17. Mol bioSystems 2012, 8, 1694-1700.

3. Pan, X., Wilson, M., McConville, C., Arvanitis, T.N., Kauppinen, R.A. and Peet, A.C. (2012) The size of cytoplasmic lipid droplets varies between tumour cell lines. MAGMA 2012 Dec;25(6):479-85. doi: 10.1007/s10334-012-0315-x.

Supplementary Tables S1 and S2

Supplementary Table 1. Single nucleotide variants with coverage >30X, MAF<0.001 and LOD > 0.5 found in the family with CANVAS. NFE, Non-Finnish European.

POS	GENE	dbSNP	Location	Function	MAF			LOD score
					1000G	GnomAD	GnomAD NFE	
chr1:38281098	MTF1	rs534357571	exonic	nonsynonymous	0.0004	0.0002	$4.5x10^{-05}$	10.616
chr1:41608664	SCMH1	rs150272371	exonic	synonymous	0.0002	0.0002	0.0004	0.8442
chr1:47746033	STIL		exonic	synonymous				0.8242
chr4:140058846	ELF2	rs747574524	exonic	nonsynonymous	0	0	0	0.5701
chr7:137776458	AKR1D1	rs150580784	intronic		0.0008	0	0	0.5402
chr11:60640710	ZP1		exonic	synonymous				0.7308
chr11:61108877	DAK	rs370562273	intronic		0	0.0002	0.0004	0.5605
chr19:9578460	ZNF560	rs146030501	exonic	nonsynonymous	0.0002	0.0004	0.0009	0.7096
chr19:9960233	PIN1	rs370315019	3’ UTR		0	$6.9x10^{-05}$	0.0001	0.6966
chr19:12921075	RNASEH2A	rs373831458	intronic		0.0006	0	0	0.5743

Supplementary Table 2. Final list of candidate variants. This list resulted after excluding non-coding (intronic, UTR3) and synonymous variants. Intronic variants were excluded after confirming that they did not generated a novel splice-site. Remaining variants were prioritized according to the combined score of four algorithms (Pathogenic variant –PAVAR- score, Exomizer, VAAST+Phevor and CADD). PAVAR score ranges from 0 to 7 and it was calculated as the sum of the tools that considered a given variant as pathogenic by using the following tools: SIFT, Polyphen2, Mutation Taster, PhyloP, GERP++, Grantham and PhastCons. Positions are related to the "Genome Reference Consortium GRCh37".

			Single Scores							Combined scores			
**Gene**	**Pos.**	**rsID**	**SIFT**	**Polyphen2**	**Mutation Taster**	**PhyloP**	**GERP++**	**Grantham**	**PhastCons**	**PAVAR**	**Exomiser**	**VAAST+ Phevor**	**CADD**
ELF2	chr4:140058846	rs747574524	0.066	0.996	1.000	4.175	5.17	58	1.000	5	0	6.17	23.3
MTF1	chr1:38281098	rs534357571	0.57	0.0	0.534	2.347	5.03	27	1.000	3	0.043	5.45	0.01
ZNF560	chr19:9578460	rs146030501	0.63	0.028	1.000	-3.915	-3.74	98	0.000	2	0	4,2	3.3
