# Supplementary material for: Clinical and Functional Characterization of a Missense ELF2 Variant in a CANVAS Family
Source: Front Genet. 2018 Mar 23;9:85. doi: 10.3389/fgene.2018.00085 (PMC5876245; doi:10.3389/fgene.2018.00085)
Supplement: Supplementary file 2 [file Image_1.pdf]

Supplementary Material

Clinical and functional characterization of a novel missense ELF2 variant in a CANVAS family

Hena Ahmad,MD[1], Teresa Requena, PhD[2], Lidia Frejo[2], Marien Cobo, PhD[3], Alvaro Gallego-Martinez[2], Francisco Martin, PhD[3], Jose A Lopez-Escamez, PhD, MD[2,4,] *, Adolfo M. Bronstein, PhD,MD[1,] *

First Author*, Co-Author, Co-Author

*** Correspondence:** Corresponding Authors: a.bronstein@imperial.ac.uk & antonio.lopezescamez@genyo.es

Supplementary Figures

```
ELF2_HUMAN      GGCAACCATCAGTGAACCTTTAATTTC--ATTGATTAATAGCGTTTGAAGCTTCC-TCAG
ELF2_PANTRE     GGCAACCATCAGTGAACCTTTAATTTC--ATTGATTAATAGCGTTTGAAGCTTCC-TCAG
ELF2_MOUSE      GGCAATCGTCTGTGAGCCTT-AGTTCC--GTTGCTTCACAGCATTTGAGACTTCCTCAGG
ELF2_XENTR      ------------------------------------------------------------
ELF2_DANRE      GT----GGGGTATGAGTCATGACTTCTGTGGTGCTTGTAGACAGTGGAGGAGCAGTGGT-

ELF2_HUMAN      GGAATAACAATGACATCAACAGTGGTTGACAGTGGAGGTACTATTTTGGAGCTTTCCAGC
ELF2_PANTRE     GGAATAACAATGACATCAGCAGTGGTTGACAGTGGAGGTACTATTTTGGAGCTTTCCAGC
ELF2_MOUSE      GAACGCACGATGGCATCGGCGGTGGTTGACAGCGGAGGCTCTGCTCTGGAGCTTCCTAGC
ELF2_XENTR      ------------------------------------------------------------
ELF2_DANRE      ----GGAGTATGTC-ACTGCAGTCGATGACCATTTACCGGAGGAGGGTGGCGTGTATGAG

ELF2_HUMAN      AATGGAGTAGAAAATCAAGAGGAAAGTGAAAAGGTTTCTGAATATCCAGCAGTGATTGTG
ELF2_PANTRE     AATGGAGTAGAAAATCAAGAGGAAAGTGAAAAGGTTTCTGAATATCCAGCAGTGATTGTG
ELF2_MOUSE      GATGGAGGAGAAAATCAAGAGGGAGGTGACACGGGCCCTGACTGCCCGGCAGTGATTGTG
ELF2_XENTR      ------------------------------------------------------------
ELF2_DANRE      GTGGAGGGTGAAATGGAGGGAGAGGTGGAGGGCGATGTGGAGTATCCAGCAGTGATTGTG

ELF2_HUMAN      GAGCCAGTTCCAAGTGCCAGATTAGAGCAGGGCTATGCAGCCCAGGTTCTGGTTTATGAT
ELF2_PANTRE     GAGCCAGTTCCAAGTGCCAGATTAGAGCAGGGCTATGCAGCCCAGGTTCTGGTTTATGAT
ELF2_MOUSE      GAGCCAGTTCCCAGTGCGAGGTTAGAGCAGGGCTATGCTGCCCAGGTGCTGGTCTATGAC
ELF2_XENTR      -------GTCTGAATCTCCGCTTCC---TCCTCTCTCCTGCCAATGTGAAGCTGAACCTC
ELF2_DANRE      GAGCCAGTGCCCAGCGCGCGGATGGAGCAAGGCTTTGCTGCTCAGGTGCTGGTGTACGAT
                         *  *      *  *         ** * * **  * **   * *  *

ELF2_HUMAN      GATGAGACTTATATGATGCAAGATGTGG---CAGAAGAACAAGAAGTTGAGACCGAGAAT
ELF2_PANTRE     GATGAGACTTATATGATGCAAGATGTGG---CAGAAGAACAAGAAGTTGAGACCGAGAAT
ELF2_MOUSE      GACGAGACTTATATGATGCAGGATGTGG---CGGAAGAACAGGAAGTTGAGACGGAGAAT
ELF2_XENTR      CGGGAT--AAATATGGCGACTTCGCTGCATGAGGGACCTACCAACCAGCTTGATCTGCTC
ELF2_DANRE      GATGAGACATACCTGATGCAGGGAGTAGCTGAGG---AGCAGGAGGTGGAGACAGAGGTG
                   **     *  **  *       *       *         *            *

ELF2_HUMAN      GTGGAAACAGTGGAAGCATCAGTTCACAGCAGTAATGCACACTGTACAGATAAGACAATT
ELF2_PANTRE     GTGGAAACAGTGGAAGCATCAGTTCATAGCAGTAATGCACACTGTACAGATAAGACAATT
ELF2_MOUSE      TCAGAAACAGTGGAAGCATCAGTTCACAGCAGTAATGCTCACTGTACGGATAAGACCATT
ELF2_XENTR      ATCAGAGCCGTGGAAGCATCGGTTCATGGCAGCAATGTTCACTGCACGGATAAGACTATT
ELF2_DANRE      CTGGAGACAGTGGAAGCATCAGTTC------ATGGTGTGCAATGCTCTGATAAAACCATC
                       * *********** ****          **  ** **  * ***** ** **
```

Supplementary Figure 1. ELF2 gene DNA sequence compared across species showing the base change. The ELF2 gene is conserved in human, chimpanzee, dog, cow, rat, chicken, and zebrafish. The variant chr4: g.140058846 C>T, c.10A>G.

```
SP|Q15723|ELF2_HUMAN   MTSAVVDSGGTILELSSNGVENQEES-EKVSEYPAVIVEPVPSARLEQGYAAQVLVYDDE 59
TR|H2QQ65|H2QQ65_PANTR MTSAVVDSGGTILELSSNGVENQEES-EKVSEYPAVIVEPVPSARLEQGYAAQVLVYDDE 59
SP|Q9JHC9|ELF2_MOUSE   MASAVVDSGGSALELPSDGGENQEGG-DTGPDCPAVIVEPVPSARLEQGYAAQVLVYDDE 59
TR|Q5XHJ7|Q5XHJ7_XENLA MTSAAVDGGGSVGEFVSNGVEKLEQEEVQVTEYPAVIVEPVPSARLEQGYAAQVLVYDDE 60
TR|Q6PHH1|Q6PHH1_DANRE ------------------------------------------------------------

SP|Q15723|ELF2_HUMAN   TYMMQDVAEEQE-VETENVETVEASVHSSNAHCTDKTIEAAEALLHMESPTCLRDSRSPV 118
TR|H2QQ65|H2QQ65_PANTR TYMMQDVAEEQE-VETENVETVEASVHSSNAHCTDKTIEAAEALLHMESPTCLRDSRS-- 116
SP|Q9JHC9|ELF2_MOUSE   TYMMQDVAEEQE-VETENSETVEASVHSSNAHCTDKTIEAAEALLHMESPTCLRDSRSPV 118
TR|Q5XHJ7|Q5XHJ7_XENLA TFMMQDVAEEHE-VETESVEIVEASVHGSNVHCTDKTIEAAEALLHMESPTILRDTRSPV 119
TR|Q6PHH1|Q6PHH1_DANRE --MATSLHEGPANQLDLLIRAVEASVH--GVQCSDKTIEAAEALLHMDSPSSLRGDRSP- 55
                         *  .: *          . ******  ..:*:*************:**: **. **

SP|Q15723|ELF2_HUMAN   EVFVPPCVSTPEFIHAAMRPDVITETVVEVSTEESEPMDTSPIPTSPDS--HEPMKKKKV 176
TR|H2QQ65|H2QQ65_PANTR ----------PEFIHAAMRPDVITETVVEVSTEESEPMDTSPIPTSPDS--HEPMKKKKV 164
SP|Q9JHC9|ELF2_MOUSE   EVFVPPCISTPEFIHAAMRPDVITETVVEVSTEESEPMDASPIPTSPDS--HEPMKKKKV 176
TR|Q5XHJ7|Q5XHJ7_XENLA EVYVSPCVSTPEFIHAAMRPDVITETVVEVSTEDSEPMDSTSIPISIEL--GEPMKKKKA 177
TR|Q6PHH1|Q6PHH1_DANRE EVFVPPCVNTSEFLHAAMRPDVLTETVVEVSTEDMEPMEVVTVIQEPEMLDTEPSKRRKS 115
                                  **:********:**********: ***:   :  .  :    ** *::*

SP|Q15723|ELF2_HUMAN   GRKPKTQQSPISNGSPELGIKKKPREGKGNTTYLWEFLLDLLQDKNTCPRYIKWTQREKG 236
TR|H2QQ65|H2QQ65_PANTR GRKPKTQQSPISNGSPELGIKKKPREGKGNTTYLWEFLLDLLQDKNTCPRYIKWTQREKG 224
SP|Q9JHC9|ELF2_MOUSE   GRKPKTQQSPVSNGSPELGIKKKAREGKGNTTYLWEFLLDLLQDKNTCPRYIKWTQREKG 236
TR|Q5XHJ7|Q5XHJ7_XENLA GRKPKMQQPAFSNGSPELGIKKKQREGKGNTTYLWEFLLDLLQDKNTCPRYIKWTQREKG 237
TR|Q6PHH1|Q6PHH1_DANRE GRKPKP--HHISNGSPDMGIKKKSREGKGS-TYLWEFLLDLLQDKNTCPRYIKWTQREKG 172
                       *****     .*****::***** *****. *****************************

SP|Q15723|ELF2_HUMAN   IFKLVDSKAVSKLWGKHKNKPDMNYETMGRALRYYYQRGILAKVEGQRLVYQFKDMPKNI 296
TR|H2QQ65|H2QQ65_PANTR IFKLVDSKAVSKLWGKHKNKPDMNYETMGRALRYYYQRGILAKVEGQRLVYQFKDMPKNI 284
SP|Q9JHC9|ELF2_MOUSE   IFKLVDSKAVSKLWGKHKNKPDMNYETMGRALRYYYQRGILAKVEGQRLVYQFKDMPKNI 296
TR|Q5XHJ7|Q5XHJ7_XENLA IFKLVDSKAVSRLWGKHKNKPDMNYETMGRALRYYYQRGILAKVEGQRLVYQFKEMPKNI 297
TR|Q6PHH1|Q6PHH1_DANRE IFKLVDSKAVSKLWGKHKNKPDMNYETMGRALRYYYQRGILAKVEGQRLVYQFKEMPKDI 232
                       ***********:******************************************:***:*

SP|Q15723|ELF2_HUMAN   VVIDDDKSETCNEDLAGTTDEKSLERVSLSAESLLKAASSV----------RSGKNSSPI 346
TR|H2QQ65|H2QQ65_PANTR VVIDDDKSETCNEDLAGTTDEKSLERVSLSAESLLKAASSV----------RSGKNSSPI 334
SP|Q9JHC9|ELF2_MOUSE   VVIDDDKSETCPEDLAAAADDKSLERVSLSAESLLKAATAV----------RGGKNSSPL 346
TR|Q5XHJ7|Q5XHJ7_XENLA VVIEEDKAEPCSEEMVSPIDDKSLERVSIPTEGIMKMAMPA----------RLEKSPTKQ 347
TR|Q6PHH1|Q6PHH1_DANRE VVIDDDKCDPGDDVI----GEKTYERVPPSSDTLLTDLSKTPTILRAADRTVLHPGSPKA 288
                       ***::**.:   : :    .:*: ***   :: ::.    .              .

SP|Q15723|ELF2_HUMAN   NCSRAEKGVARVVNITSPGHDASSRSPTTTASVSATAAPRTVRVAMQVPVVMT-SLGQKI 405
TR|H2QQ65|H2QQ65_PANTR NCSRAEKGVARVVNITSPGHDASSRSPTTTASVSATAAPRTVRVAMQVPVVMT-SLGQKI 393
SP|Q9JHC9|ELF2_MOUSE   NCSRAEKGVARVVNITSPTHDGSSRSPTTTAPVSAAAAPRTVRVAMQVPVVMT-SLGQKI 405
TR|Q5XHJ7|Q5XHJ7_XENLA SSNR----KPRLVSWSCTANEASPHS-----PTSTTPTQRTVRVAMQVPVVMT-SLGQKI 397
TR|Q6PHH1|Q6PHH1_DANRE KAALSATPVQRTV-------MVSTADPSHATIISNANAPRTVRVAMQVPVVMTNSLGQKI 341
                       ..        * *         *  .       * : : ************** ******

SP|Q15723|ELF2_HUMAN   STVAVQSVNA--GAPL-ITSTSPTTATSPKVVIQTIPTVMPASTENGDKITMQPAKIITI 462
TR|H2QQ65|H2QQ65_PANTR STVAVQSVNA--GAPL-ITSTSPTTATSPKVVIQTIPTVMPASTENGDKITMQPAKIITI 450
SP|Q9JHC9|ELF2_MOUSE   SAVAVQSVNAGTGSPL-ITSTSPASASSPKVVIQTVPTVMPASTENGDRITMQPAKIITI 464
TR|Q5XHJ7|Q5XHJ7_XENLA STLAVQSMNP--GSPL-IANSSPTTASAPKVVIQTIPTMLPASSESGDKFTMQPAKIITI 454
TR|Q6PHH1|Q6PHH1_DANRE STVAVQSTNPSLLTTAPTNTGSPTGTNAPKVLIQTMPTMVPATAENGDKITVQLAKIITI 401
                       *::**** *    :     . **: :.:***:***:**::**::*.**::*:* ******

SP|Q15723|ELF2_HUMAN   PATQLAQCQLQTKSNLTGSGSINIVGTPLAVRALTPVSIAHGTPVMRLSMPTQ-QASG-- 519
TR|H2QQ65|H2QQ65_PANTR PATQLAQCQLQTKSNLTGSGSINIVGTPLAVRALTPVSIAHGTPVMRLSMPTQ-QASG-- 507
SP|Q9JHC9|ELF2_MOUSE   PATQLAQCQLQAKSNLTGSGSINIVGTPLAVRALTPVSIAHGTPVMRLSVPAQ-QASG-- 521
TR|Q5XHJ7|Q5XHJ7_XENLA PTSQLTQCQLQTKPGLSGSGGLNLVGTPLTVRALTPVSIAHGTPVMRLAMPAAHQARC-- 512
TR|Q6PHH1|Q6PHH1_DANRE PATQLTQCQLQAKPGT--PTGINLMGAPLTVRALTPMSVAPGTQVVRLAVPAQQSPVQAK 459
                       *::**:*****:* .     .:*::*:**:******:*:* ** *:**::*:  .

SP|Q15723|ELF2_HUMAN   -QTPPRVISAVIKGPE-VKSEAVAKK--QEHDVKTLQLVE-EKPADGNKTVTHVVVVSAP 574
TR|H2QQ65|H2QQ65_PANTR -QTPPRVISAVIKGPE-VKSEAVAKK--QEHDVKTLQLVE-EKPADGNKTVTHVVVVSAP 562
SP|Q9JHC9|ELF2_MOUSE   -QTPPRVISALLKGPE-GKSE--AKK--QEHDVKTLQLVE-EKGADGNKTVTHVVVVSAP 574
TR|Q5XHJ7|Q5XHJ7_XENLA -QTPPRVISTLIKGPE-IKSDMVVGR--QEGELKTFQFIKDENPADGGKTVTHVVVVSTP 568
```

Supplementary Figure 2. elf-2 protein sequence compared across species showing the amino acid change. The elf-2 gene is conserved in human, chimpanzee, dog, cow, rat, chicken, and zebrafish. The position p.Ala4 is indicated with an arrow.

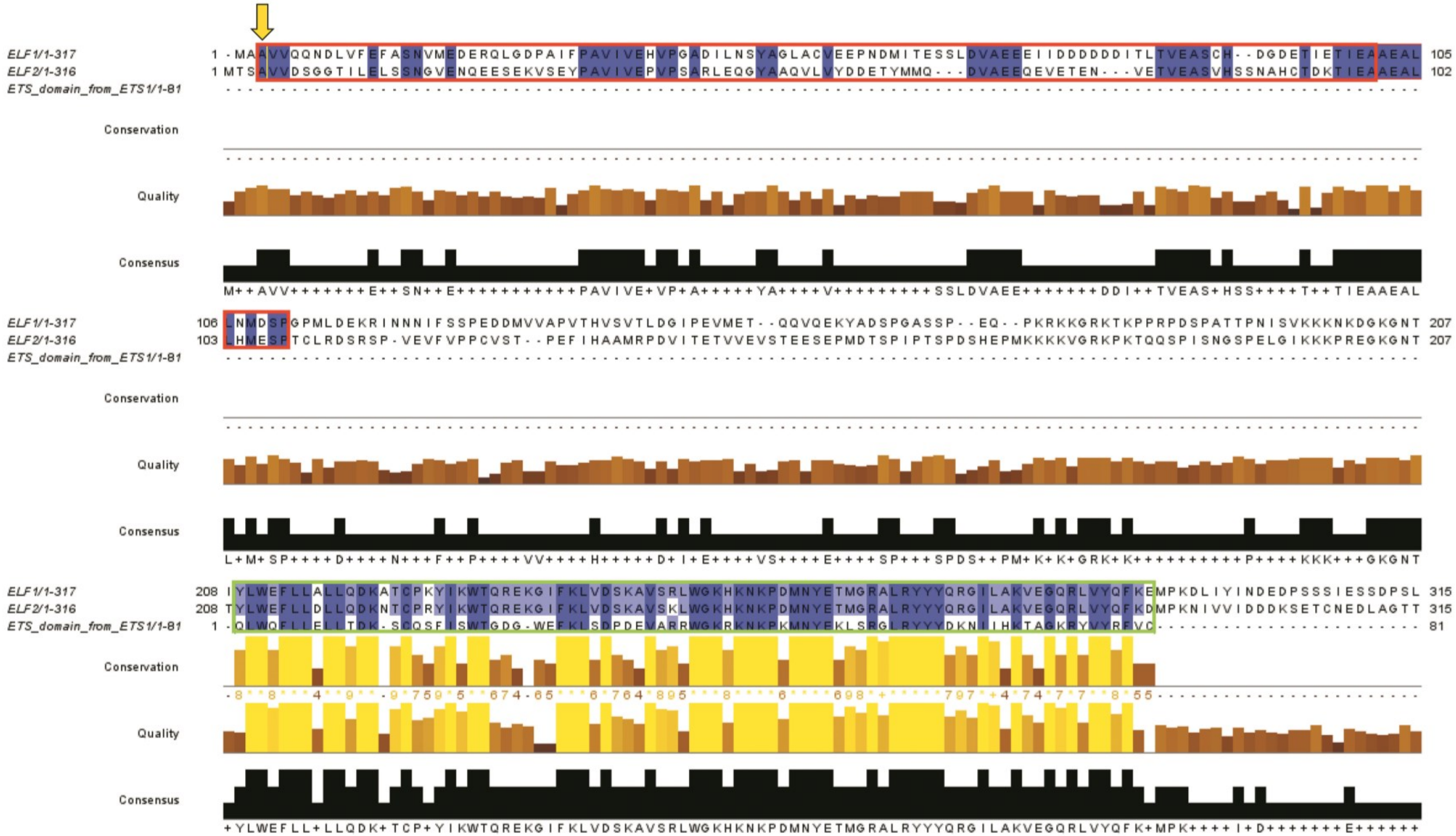

Supplementary Figure 3. Alignment between ELF2 protein sequence, ELF1 protein sequence and ETS domain sequence from ETS1 protein sequence. Alignment was made with ClustalO tool and managed in JalView Software. The relevant position in our study is marked with a yellow arrow at the beginning of the alignment (p.Ala4). This position is highly conserved between three sequences. ETS domain sequence alignment is marked in green box. ELF-1 transcription binding domain is marked in a red box. Highly conserved amino acids between sequences in both domains are highlighted in dark blue.

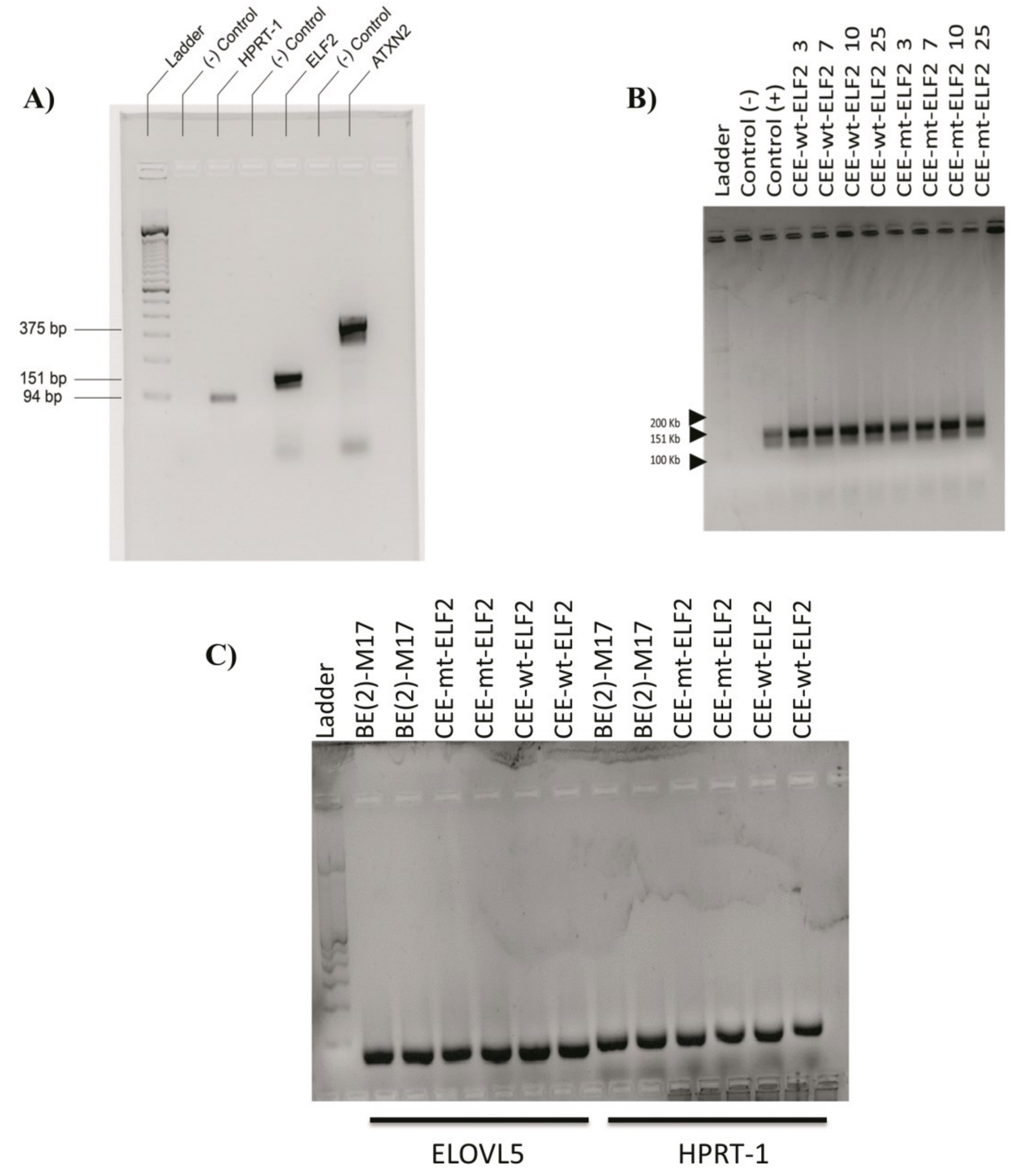

Supplementary Figure 4. A) Constitutive expression of ELF2 and ATXN2 genes in the neuroblastoma cell-line BE (2) M17. B) PCR of CEE-mt-ELF2 and CEE-wt-ELF2 in BE(2)M17 cells over time. Vector remained stable at day 3, 7, 10 and 25 after transduction. C) Constitutive expression of ELOVL5 and HPRT1 genes in BE (2) M17 cells.

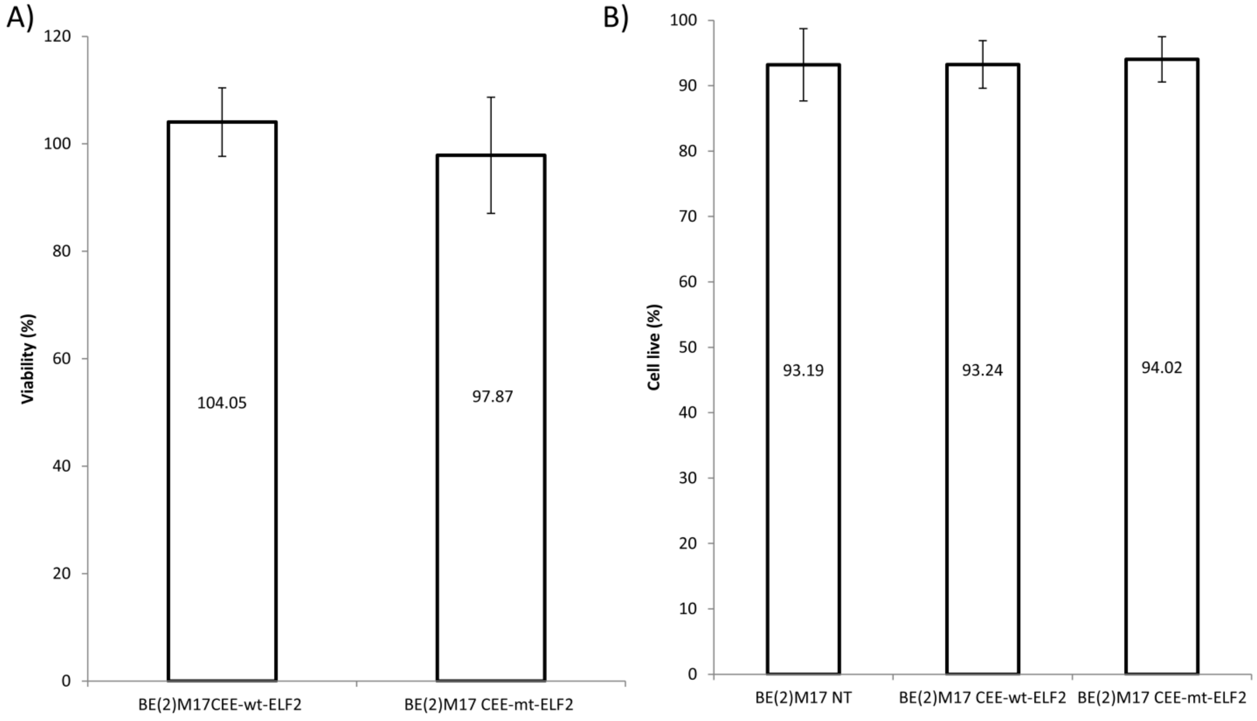

Supplementary Figure 5. Viability after transduction. The cell-line BE(2)M17 was transduced with CEE-mt-ELF2 and CEE-wt-ELF. Proliferation and survival rate were measured at 24h, 48h, 3, 7,10 and 14 days. Viability after transduction was measure by A) mitochondrial activity (WST-1 assay) and B) percentage of live and dead cells.
